# Robust cross-immunity against a heterologous field strain of foot-and-mouth disease virus achieved in small ruminants following commercial vaccination in Jordan

**DOI:** 10.3389/fvets.2026.1872333

**Published:** 2026-07-27

**Authors:** Efrem A. Foglia, Giampietro Maccabiani, Cornelis van Maanen, Vito Tranquillo, Emad M. Bennour, Hashim A. Mansour, Nussieba A. Osman, Mohammad Khalifeh, Ausama A. Yousif, Maisa Al-Ameer, Mohammed S. Bin-Tarif, Saeda Salah, Shawkat Lafi, Zaidoun S. K. Hijazeen, Wafaa A. Ramadneh, Shahin Baiomy, Francesca Ambrosini, Donal Sammin, Fabrizio Rosso, Santina Grazioli

**Affiliations:** 1 Istituto Zooprofilattico Sperimentale Della Lombardia e dell’Emilia-Romagna Bruno Ubertini, Brescia, Italy; 2 Food and Agriculture Organization of the United Nations (FAO), Rome, Italy; 3 Department of Internal Medicine, Faculty of Veterinary Medicine, University of Tripoli, Tripoli, Libya; 4 The Central Laboratory for Evaluation of Veterinary Biologics, Cairo, Egypt; 5 Department of Pathology, Parasitology and Microbiology, College of Veterinary Medicine, Sudan University of Science and Technology, Khartoum North, Sudan; 6 Department of Basic Veterinary Medical Sciences, Jordan University of Science and Technology, Irbid, Jordan; 7 Department of Virology, Faculty of Veterinary Medicine of Cairo University, Giza, Egypt; 8 Virology Unit, Animal Wealth Laboratories Directorate, Ministry of Agriculture, Amman, Jordan; 9 Livestock Department, Ministry of Agriculture, Amman, Jordan; 10 Early Warning and Risk Assessment Department, Veterinary and Animal Health Directorate, Ministry of Agriculture, Amman, Jordan; 11 Department of Veterinary Pathology and Public Health, Faculty of Veterinary Medicine, Jordan University of Science and Technology, Irbid, Jordan; 12 National Project Personnel, Animal Health, Food and Agriculture Organisation of the United Nations, Rome, Italy; 13 NPO Programme, Food and Agriculture Organisation of the United Nations, Amman, Jordan

**Keywords:** field studies, foot and mouth disease, vaccine, vaccine evaluation, veterinary vaccination, small ruminants

## Abstract

**Introduction:**

Foot-and-mouth disease (FMD) is a contagious infectious disease, with a severe socio-economic impact, affecting cloven-hoofed livestock. Routine and emergency vaccinations are the most effective tool for the containment and the eradication of the disease in endemic countries. Due to the presence of the virus in seven serotypes and in various lineages and strains, the selection of the most appropriate vaccine is essential to achieve protection. According to WOAH/FAO guidelines, assessing vaccine effectiveness is recommended to improve control strategies. This study reports the analysis of the results from Post Vaccination Monitoring (PVM) performed in Jordan across 2022 and 2023.

**Methods:**

A polyvalent inactivated vaccine, including one strain serotype O (O/EURO-SA/O_1_/Campos) and two strains serotype A (A/EURO-SA/A_24_/Cruzeiro and A/ARG/2001), was administered to naïve small ruminants (goats and sheep). About one half of the animals received a second dose 28 days after the first. Antibody titres were determined by Virus Neutralisation Test (VNT) against two FMD serotype O strains: one closely related to the vaccine strain and the other that had recently circulated in the country.

**Results:**

Overall, a single administration of the vaccine was effective in inducing a good response in small ruminants, but a second administration boosted immunity, increasing the level and duration of the immune response in almost all vaccinated animals. The same vaccinal response was apparent when measured against the field strain.

**Discussion:**

This field study corroborated the results of vaccine matching and confirmed the current suitability of that particular vaccine formula in the containment of FMD in the Middle East area.

## Graphical abstract

**Figure:**
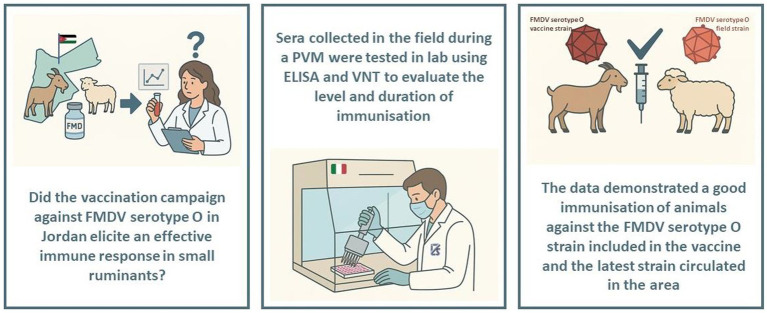

## 1 Introduction

Foot-and-mouth disease (FMD) is a highly contagious infectious disease affecting wild and domestic cloven-hoofed animals. The causative agent of the disease is *Aphthovirus vesiculae* (Foot-and-mouth disease virus–FMDV) of the genus *Aphthovirus*, family *Picornaviridae* (1). The virus is highly contagious and may circulate widely in affected areas through direct contact between animals, contact with contaminated objects, aerosols, and consumption of contaminated products, leading to detrimental socio-economic consequences (2–4). In endemic countries, costs related to FMD outbreaks have been estimated between US$ 6.5 and 21 billion per annum, and even countries that are FMD-free still incur indirect expenses exceeding US$ 1.5 billion (3). This considerable economic impact is due to many factors; such as the decrease in livestock production, the reduced access of affected countries to global markets, the procedures needed to contain virus circulation and to gain, re-gain, or maintain the FMD-free status (3). In the African and Asian continents, the disease circulates endemically, while in South America some occasional recurrences have been recorded (5). The circulation of the disease in the Middle East, in Anatolia (Türkiye) and in North Africa has always been considered as a continuous threat to those countries where FMD had been eradicated and the recent reappearance of the disease in some European countries (i.e., Germany, Hungary and Slovakia; 2025) (5) confirmed that this risk is serious. The menace of FMD spread to disease-free countries necessitates regional and international cooperation to implement effective disease prevention and control measures.

Owing to its strategic location, the Hashemite Kingdom of Jordan may play a key role in monitoring the circulation routes of different FMDV lineages in the Middle East and in controlling the geographical spread of the disease from Asia toward neighbouring European and North African countries and vice versa. Jordan, where FMD is endemic (6, 7), is bordered by other FMD-endemic countries, namely Syria to the North, Iraq to the East, Israel and Palestine territories to the West,^1^ and the Kingdom of Saudi Arabia (KSA) to the South, which is currently in Stage 2 of the Progressive Control Pathway (PCP-FMD), according to FAO (Food and Agriculture Organization) and WOAH (World Organization for Animal Health) (8).^2^ The latest FMDV lineages that have circulated in Jordan include serotype SAT2 (SAT2/XIV) in 2023 (9) and serotype O (O/ME-SA/PanAsia-2/ANT-10) in 2021 (10).

Vaccination is the principal prophylactic tool for controlling FMD outbreaks and containing FMDV spread or sporadic incursion from endemic regions. As a result, approximately 2.5 billion vaccine doses are administered globally to livestock each year (3, 11). The selection of the most effective vaccine formula remains a challenge because of the existence of seven serotypes of FMDV (O, A, C, Asia1 and Southern African Territory [SAT] 1–3; serotype C has not been officially reported in a confirmed global outbreak for over 21 years) (12), and this complexity is further compounded by the high antigenic variability of the circulating lineages and the constant emergence of new variants driven by evolutionary pressure (2, 13, 14). There are many commercial vaccines available against FMD but choosing the most suitable for any given area will depend on the virus strains in circulation (15). Thus, the essential step in vaccine selection is evaluating the antigenic correspondence between the FMDV strain of interest for the region in question (the field strain) and the strain(s) incorporated into the vaccine. This information is usually determined by vaccine matching (16), but further field investigations may help to evaluate the likely success of vaccination, involving in the evaluation also other factors, such as the assessment of cold-chain maintenance, the accuracy of administration or the duration of induced immunity in animals (1, 17). Small-scale vaccination trials and Post-Vaccination Monitoring (PVM) are powerful tools, together with vaccine matching, to estimate the likely efficacy of FMD vaccines (11) and predict the overall effectiveness of a vaccination campaign (18–21).

Because of the crucial role of vaccination in FMD control, FAO and WOAH drew up specific guidelines for PVM (22) and in several countries, including Jordan, programs to support local vaccination campaigns are promoted by The European Commission for the control of Foot-and-Mouth Disease (EuFMD).

This study describes the results of a PVM conducted in Jordan across 2022 and 2023, promoted by EuFMD and performed following the FAO and WOAH guidelines (22). The project focused on the immunisation of small ruminants against FMD serotype O virus following vaccination with a trivalent commercial vaccine, with particular emphasis on the efficacy against the FMDV strain circulating in the region. The efficacy of the vaccine, selected for the Jordan campaign, to induce an immunisation was evaluated by measuring neutralising antibodies, that arose after vaccination, and comparing the results with other studies (i.e., vaccine matching). A serological analysis was undertaken (23), based on Virus Neutralisation Test (VNT), determining the presence of antibodies able to neutralise an FMDV strain phylogenetically close to that included in the vaccine and a serotype O strain currently circulating in Jordan and still threatening the country.

To date, several studies have been conducted to predict and evaluate the ability of this commercial vaccine to induce effective cross-neutralisation against FMDV serotype O strains circulated in Asia (24–26). However, the presented study is the first to analyse field data obtained in Middle East for the evaluation of the vaccine’s suitability in that area. This approach enabled a more reliable evaluation of the effectiveness of the selected vaccine.

## 2 Materials and methods

### 2.1 Ethical statement

The General Secretary Assistant for Livestock Affairs of the Hashemite Kingdom of Jordan had an agreement with local farmers or stakeholders to vaccinate animals and collect samples, certifying the respect of WOAH standards for animal welfare (doc. 5/5/15, 2024/12/23).

### 2.2 Animals

**Figure 1:**
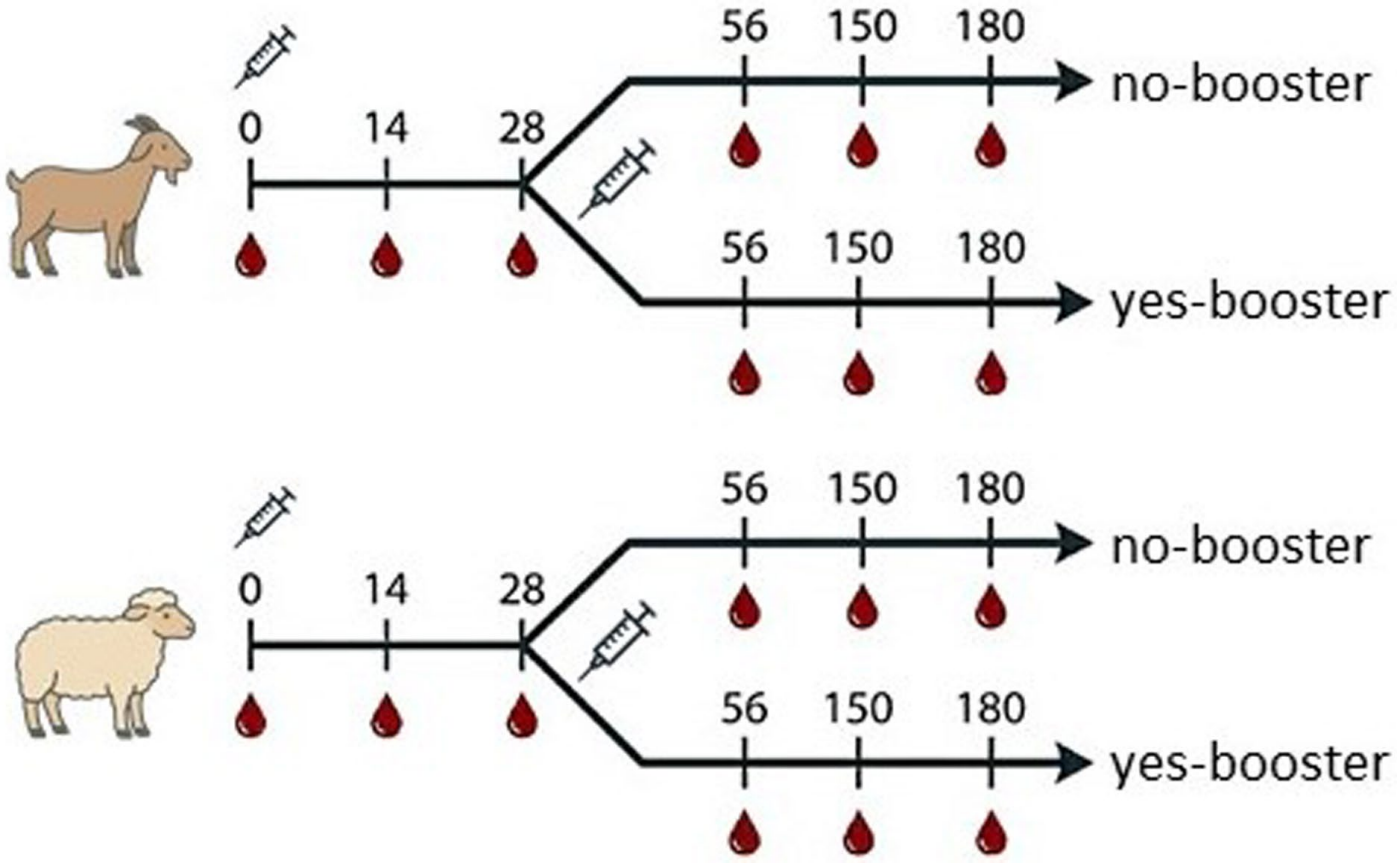
Scheme of vaccination and samplings. Graphical representation summarizing the design of the study involving two populations of small ruminants, goats (above) and sheep (below). The two timelines report the points of vaccination (syringe) and of blood samplings (red drops) from day 0 to 180 DPV. At 28 DPV one subgroup of each population received a booster vaccination and each timeline splits into two: the upper lines represent single vaccinated animals (no-booster) while the lower lines indicate double-vaccinated animals (yes-booster).

According to the experimental design suggested by FAO/WOAH for PVM studies (22), which recommends to involve at least 20 animals for each group, 116 small ruminants (60 goats and 56 sheep) were enrolled in the study. All small ruminants were between 6 and 12 months of age, had never been exposed to FMDV infection (non-vaccinated and seronegative for antibodies against non-structural proteins of FMDV (FMDV-NSP)), and without structural proteins-specific (SP-specific) maternally-derived antibodies from vaccinated or infected dams. Animals were individually labelled and divided into two groups: the first (33 goats and 30 sheep) received a single dose of vaccine at day zero; the second (27 goats and 26 sheep) received two doses of vaccine, one at day zero and a second (booster) dose at day 28 (Figure 1).

Animals were excluded from the study if they tested positive for antibodies against NSP-FMD (indicating a suspected previous infection) or if they tested positive for antibodies against SP-FMD prior to vaccination (indicating suspected maternally derived antibodies).

Experimental animals were kept in two distinct areas located approximately 140 km apart: goats in Wala area, located in Madaba Governorate along the Wadi Al-Wala watershed, and sheep in Khanasari area, located in Mafraq Governorate. Despite the fact that the two regions experienced circulation of the same FMDV serotypes and strains, no data on variables that may influence immune response were collected. Variations related to climate, temperature, diet, and housing may influence the stress level and immune status of the animals (27, 28). Consequently, the comparison between the two groups could not be as thorough as desired, limiting the study to the mere observation of any differences between the two groups.

### 2.3 Vaccine

Vaccination was performed using the trivalent vaccine BIOAFTOGEN®, a water-in-oil single emulsion vaccine manufactured by Biogénesis Bagó (Garín, Buenos Aires, Argentina). According to the manufacturers’ information, the vaccine provides at least 6 PD_50_ per dose (1 mL) of each of the following viruses, O/EURO-SA/O_1_/Campos, A/EURO-SA/A_24_/Cruzeiro and A/ARG/2001. The vaccine was produced starting from FMDV strains cultured on a suspension of BHK cell line (ATCC CCL-10), inactivated with binary ethylenimine (BEI) and purified before blending. The declared shelf life of the vaccine was 24 months and it was stored at a temperature of 2–8 °C.

### 2.4 Sampling scheme

Blood samples were collected by venipuncturefrom the jugular vein at specific timepoints as depicted in (Figure 1) and according to the following scheme: Blood samples were centrifuged at 3000 g for 6 min at room temperature and 2 mL serum aliquots were stored at −20 °C.

**Table:** 

0 days post-vaccination (DPV)	Immune status before vaccination
14 days post-vaccination (DPV)	Evaluation of early immune-response to vaccine
28 days post-vaccination (DPV)	Standard time to evaluate vaccine-induced immune response
56 days post-vaccination (DPV)	Evaluation of immune response induced by booster vaccination
150 days post-vaccination (DPV)	Evaluation of the duration of the immune response
180 days post-vaccination (DPV)	Evaluation of the duration of the immune response

### 2.5 Discrepancies with the original design

Virus neutralisation at day 0 revealed that four goats and four sheep were positive against one or both of the virus strains used for the test, with low titres, suggesting the presence of residual maternally derived antibodies against FMDV serotype O, although no confirmatory tests were performed. Based on this speculation, and according with the exclusion criteria described in section 2.2, those animals were excluded by the analyses. Therefore, the number of animals in the study for which valid results were available was 56 goats and 52 sheep; of which 32 goats and 22 sheep received the booster dose of vaccine.

Because this trial was conducted under field conditions, it was not possible to sample all the animals at the last two timepoints, i.e., 150 and 180 DPV. Therefore, 23/24 single-vaccinated and 27/32 double-vaccinated goats were sampled at 150 and 180 DPV. The corresponding numbers for sheep, were 16/30 single-vaccinated and 13/22 double-vaccinated animals, but only 12 from the latter group were sampled at 180 DPV (Table 1).

**Table 1:** Number of samples, medians of VNT titres and positivity rates.

		GOATS						SHEEP					
		Single Vaccinated			Double Vaccinated			Single Vaccinated			Double Vaccinated		
Virus Strain	DPV	n. animals	Median VNT titres	Positivity Rate	n. animals	Median VNT titres	Positivity Rate	n. animals	Median VNT titres	Positivity Rate	n. animals	Median VNT titres	Positivity Rate
O BFS	0	56	1.04	0%				52	0.98	0%			
	14	56	2.65	98%				52	2.05	77%			
	28	56	3.14	100%				52	2.47	81%			
	56	24	2.85	100%	32	3.4	100%	30	2.26	79%	22	3	100%
	150	23	2.66	87%	27	3.1	96%	16	1.71	56%	13	2.34	100%
	180	23	2.34	86%	27	2.63	96%	16	1.81	60%	12	2.11	77%
O JORD	0	56	0.95	0%				52	0.92	0%			
	14	56	2.71	100%				52	2.16	76%			
	28	56	3.02	100%				52	2.02	73%			
	56	24	2.85	96%	32	3.42	100%	30	2.11	66%	22	3.01	100%
	150	23	2.18	74%	27	2.53	93%	16	1.52	53%	13	2.41	100%
	180	23	2.37	86%	27	2.67	96%	16	1.66	53%	12	2.43	100%

The table recaps the number of valid samples included in the analyses and their data obtained by VNT, i.e., the medians of titres expressed in log10 and the rate of positivity (%). The data are grouped depending on (i) the species, (ii) the number of doses received, (iii) the FMDV strain used for the test and (iv) the sampling timepoints.

### 2.6 ELISA for the detection of NSP-FMDV antibodies

All sera were tested to exclude the presence of antibodies against the NSP of FMDV using the 3ABC trapping indirect ELISA developed and validated by IZSLER (29, 30) and commercially available (FMDV 3ABC-trapping ELISA, IZSLER, Brescia, Italy). Briefly, sera were tested at 1/100 dilution against the 3ABC recombinant antigen immune-captured by a monoclonal antibody, then a peroxidase-conjugated monoclonal antibody specific for ruminant IgG was used as a detector. Results were expressed as percentage positive of the tested sera, referred to the reaction of a strong positive control serum present in each ELISA plate, used as 100%. The positivity threshold was set to 10%. All sera with an equal or higher percentage were considered as positive.

### 2.7 Virus neutralisation test

The use of VNT is advantageous to evaluate the effectiveness of a vaccine, because it detects and titrates antibodies able to neutralise virus infectivity *in vitro*, a datum most likely correlated to protection (22, 29, 30). Briefly, VNTs were carried out by testing sequential dilutions of sera in duplicates, starting from 1/16 (final serum dilution in the serum/virus mixture), against a virus dose of 100 (accepted range 30–300) Median Tissue Culture Infectious Doses (TCID_50_) using IB-RS-2 cell line (CVCL_4528) according to the method outlined in the WOAH Manual (29) and certified as ISO17025 in IZSLER laboratory. Because the PVM was focused on the evaluation of immunisation of the livestock population from FMDV serotype O, two strains of type O were used for the VN test. The first was O/EURO-SA/O_1_/BFS 1860/UK/67 (“O BFS”), belonging to the same lineage as that included in the vaccine, and the other was a strain that recently circulated in Jordan and continues to threaten the area, O/ME-SA/PanAsia-2/ANT-10/JOR/6/21 (“O JORD”). VNT titres were expressed as log_10_ of the reciprocal of the highest dilution protecting half of the inoculated cell cultures. The adopted threshold for positivity, in line with the WOAH Manual, is 1/32 (log_10_32 = 1.5); therefore, titres ≥32 (final serum dilution in the serum/virus mixture) were considered positive (29).

### 2.8 Statistical analysis

The point and interval estimate of the proportion of animals that converted on the different timepoints was obtained by the Agresti-Coull method (31), using the binom.confint() function of the binom package (32) and the results shown in graphical format using functions from the ggplot2 package (33) in the R environment (34).

Sheep and goats were analysed separately. For each species, log-transformed antibody titres at 28 days post-vaccination were compared between the two vaccine groups using an independent two-sample t-test (Welch Two Sample t-test). The two groups were considered independent. Statistical analyses were performed using R (R Foundation for Statistical Computing, Vienna, Austria).

## 3 Results

The absence of FMD clinical signs and the lack of NSP-FMDV antibodies, as determined by 3ABC-ELISA (data not shown), confirmed that all the animals enrolled in this study had not previously been exposed to live FMDV. All results pertain exclusively to animals that tested VNT negative at day 0; animals with VNT positive results were excluded from the analyses, as described before.

### 3.1 Goats

#### 3.1.1 Ability to neutralise the strain “O BFS”

**Figure 2:**
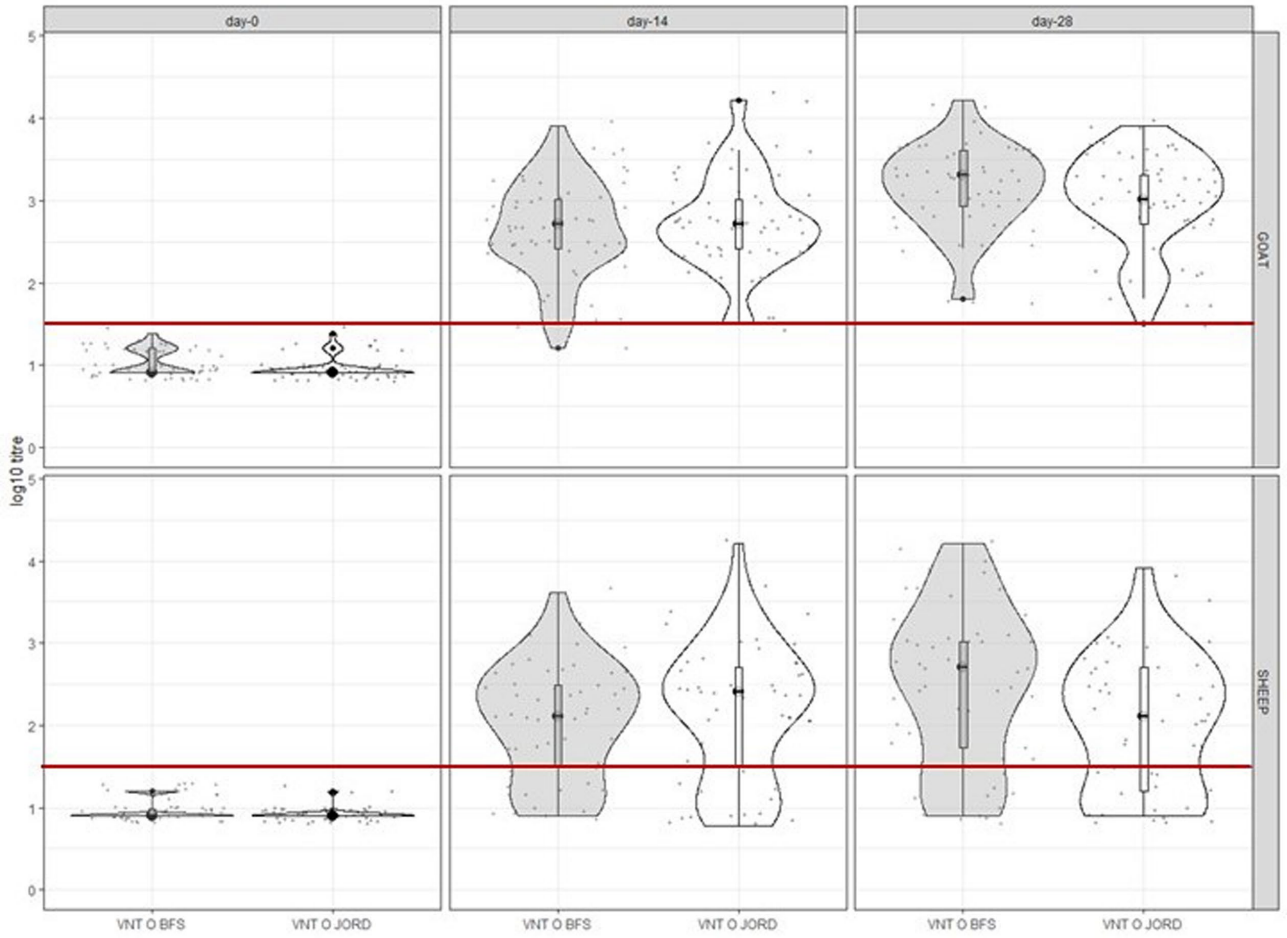
Immune status of animals after the administration of one dose of vaccine. The violin plots show the results of VNT obtained using the FMDV strain O/EURO-SA/O_1_/BFS 1860/UK/67 (O BFS, in grey) and O/ME-SA/PanAsia-2/ANT-10/JOR/6/21 (O JORD, in white) expressed as log_10_ titres. From left to right, results testing sera collected at day 0, 14 DPV and 28 DPV. Top area, VNT results obtained from goat sera; bottom area, VNT resulted from sheep sera. Every violin plot, calculated using Kernel Density Estimation (KDE), contain an inner box plot, showing the interquartile range (IQR) and the exact median (horizontal black line). The whiskers reach minimum and maximum of data, excluding outliners. The red lines indicate the VNT positivity threshold (≥ log_10_32, 1.5).

**Figure 3:**
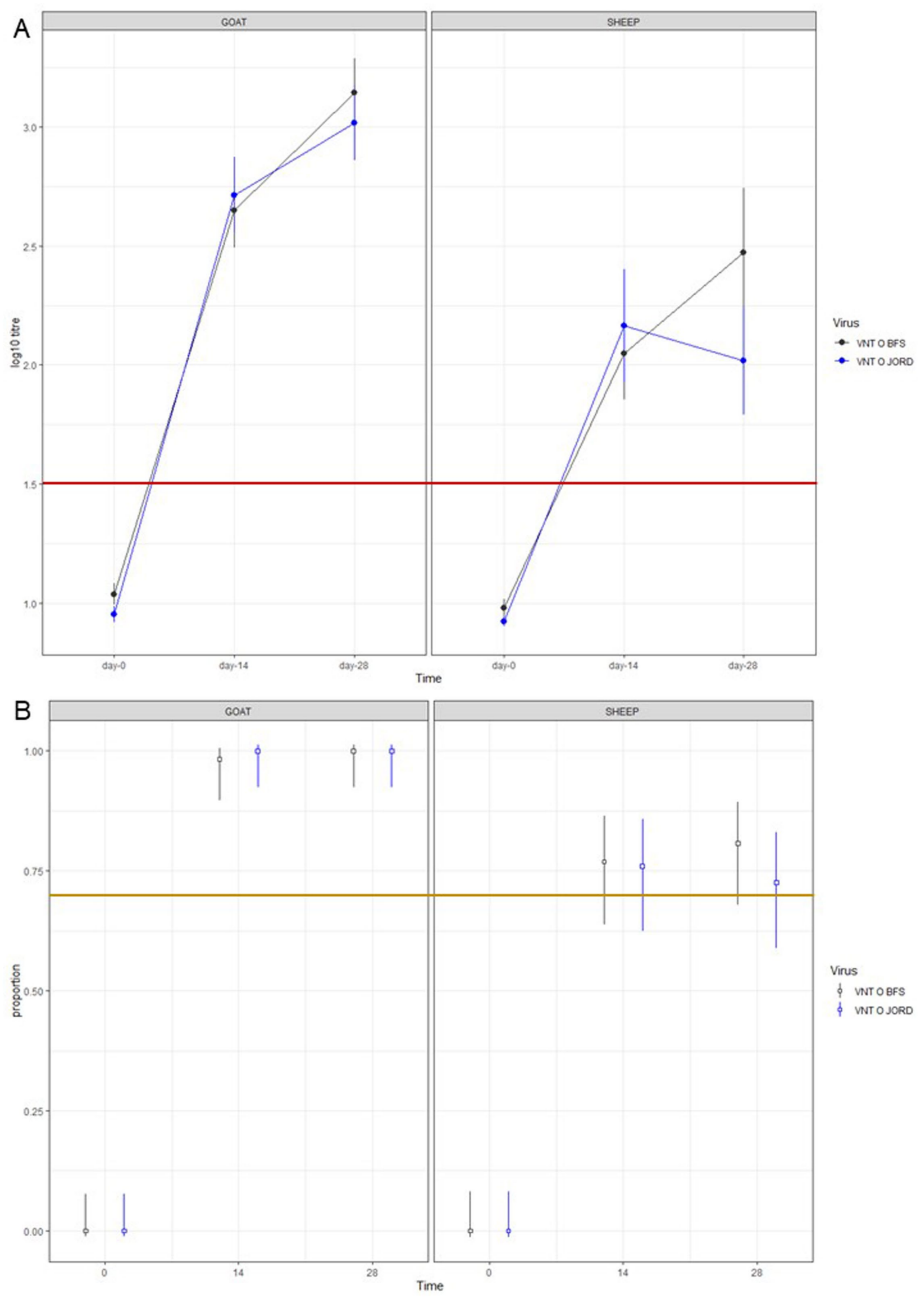
VNT titres and proportion of seropositive animals at early stages. The graphs show the immune response of animals receiving a first administration of vaccine. **(A)** Median of VNT titres, expressed as log_10_, against the FMDV strains O BFS (black line) and O JORD (blue line) using sera from goats (left) and sheep (right) at day 0, 14 DPV and 28 DPV. Error bars indicate standard deviations. The red line stands for VNT positivity threshold (≥ log_10_32, 1.5). **(B)** Proportion of seropositive animals in the population, expressed as percentage of animals testing positive for VNT against the FMDV strains O BFS (black line) and O JORD (blue line) using sera from goats (left) and sheep (right) at day 0, 14 DPV and 28 DPV. Error bars indicate 95% confidence intervals. Yellow line indicates the threshold of effectiveness of vaccination, set to 70% of a population.

**Figure 4:**
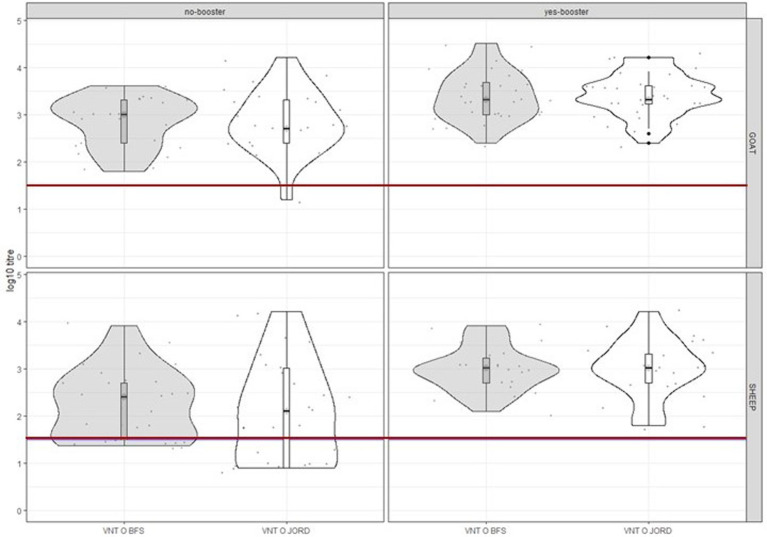
Early immunisation induced by booster vaccination. The violin plots show the results of VNT obtained testing goat (upper area) and sheep (lower area), using the FMDV strains O BFS (grey) and O JORD (white), expressed as log_10_ titres. The left section shows results testing sera from single-vaccinated animals (no-booster) and the right section from double-vaccinated animals (yes-booster). Every violin plot, calculated using KDE, contain an inner box plot, showing the IQR and the exact median (horizontal black line). The whiskers reach minimum and maximum of data, excluding outliners. The red lines indicate the VNT positivity threshold (≥ log_10_32, 1.5).

**Figure 5:**
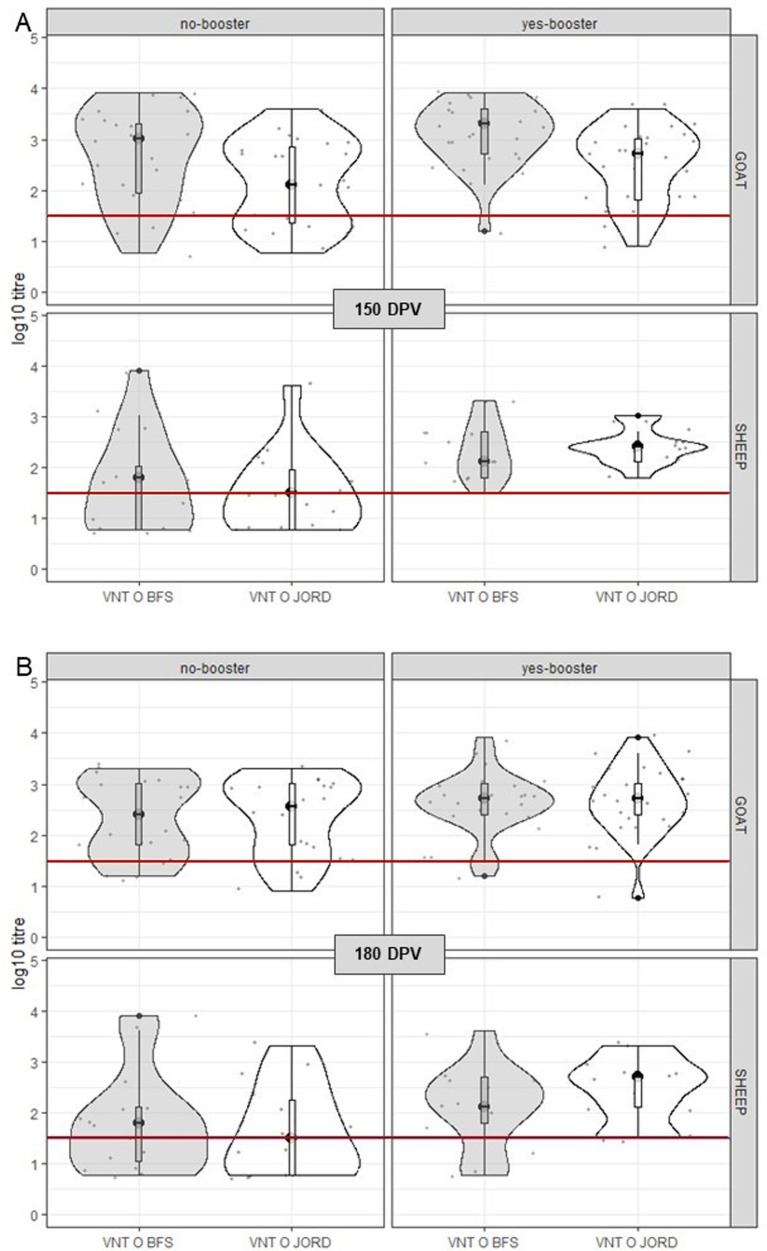
Duration of the induced immunisation in animals receiving a single dose or two doses of vaccine. The violin plots show the results of VNT obtained using the FMDV strains O BFS (grey) and O JORD (white) expressed as log_10_ titres. **(A)** Data collected at day 150 (150 DPV) and **(B)** at day 180 (180 DPV). The left section shows results from single-vaccinated animals (no-booster) and the right section from double-vaccinated animals (yes-booster). Top area, VNT results from goat sera; bottom area, results from sheep sera. Every violin plot, calculated using KDE, contain an inner box plot, showing the IQR and the exact median (horizontal black line). The whiskers reach minimum and maximum of data, excluding outliners. The red lines indicate the VNT positivity threshold (≥ log_10_32, 1.5).

**Figure 6:**
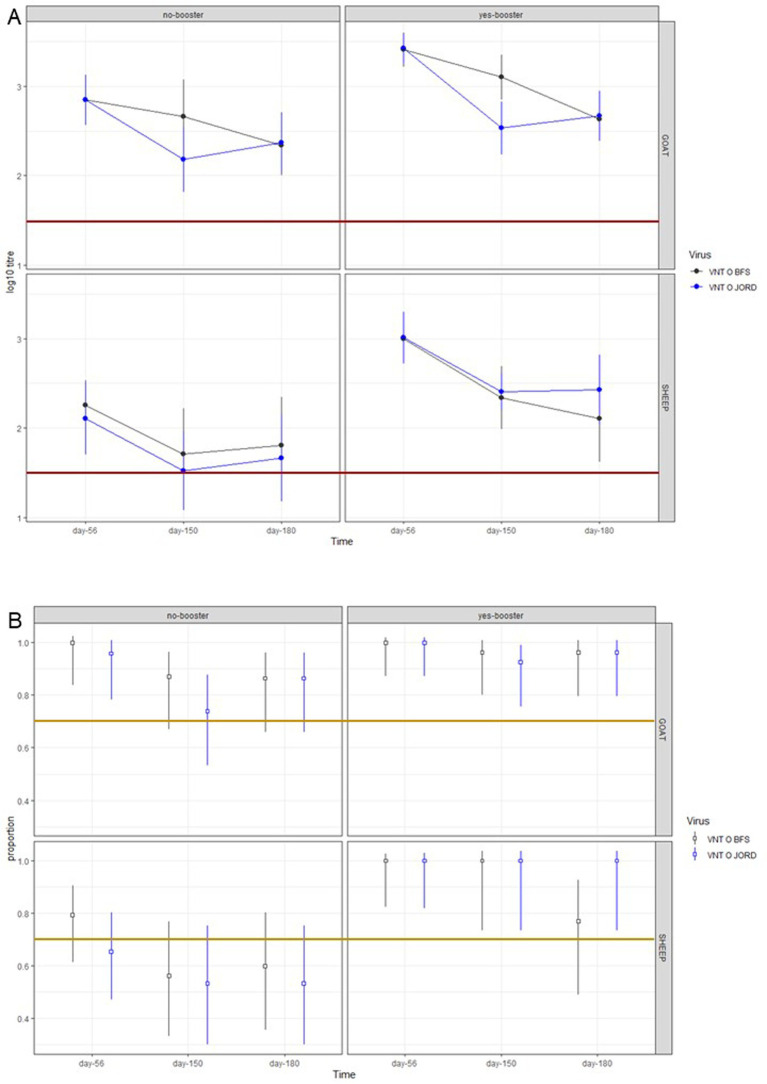
Duration of VNT titres and proportion of seropositive animals. The graphs show the immune response of the animals involved in the study. **(A)** Median of VNT titres, expressed as log_10_, against the FMDV strains O BFS (black line) and O JORD (blue line) using sera from single-vaccinated (no-booster, left) and double-vaccinated (yes-booster, right) at 56 DPV, 150 DPV and 180 DPV. Upper area, results from goat sera; lower area, from sheep sera. Error bars indicate standard deviations. The red lines indicate the VNT positivity threshold (≥ log_10_32, 1.5). **(B)** Proportion of seropositive animals in the population, expressed as percentage of animals testing positive for VNT against the FMDV strains O BFS (black line) and O JORD (blue line) using sera from single-vaccinated (no-booster, left) and double-vaccinated animals (yes-booster, right) at 56 DPV, 150 DPV and 180 DPV. Upper area, results from goat sera; lower area, from sheep sera. Error bars indicate standard deviations. Error bars indicate 95% confidence intervals. Yellow line indicates the threshold of effectiveness of vaccination, set to 70% of a population.

The administration of the vaccine to the goats induced a good immune response after a single administration, both in terms of VNT titres and percentage of VNT-positive animals (Figures 2, 3). At 14 DPV, 98% of animals were positive against the strain homologous to that contained in the vaccine, with a median VNT titres of 2.65 log_10_ and at 28 DPV, all vaccinated goats were positive (100%) with a median of 3.14 log_10_ (Figures 2, 3, Table 1). The level of immunity developed in response to a single vaccine administration reduced slightly over the course of the study. At 56 DPV, 100% of goats continued to be VNT-positive with median VNT titres of 2.85 log_10_ but only 87% of the goats remained VNT-positive at 150 DPV and 86% at 180 DPV with good median VNT titres of 2.66 log_10_ and 2.34 log_10_, respectively, (Figures 4–6; Table 1).

The 32 goats that received a second dose of vaccine, 28 days after the first dose, were all strongly VNT positive with a median VNT titre of 3.4 log_10_ at 56 DPV, 96% with a median VNT titre of 3.1 log_10_ at 150 DPV and 96% with a median of 2.63 log_10_ at 180 DPV (Figures 4–6, Table 1).

#### 3.1.2 Ability to neutralise the strain “O JORD”

Vaccination in goats also induced an effective neutralising antibody response against the strain O JORD. A single administration of vaccine induced a strong immune response (median VNT titres of 2.71 log_10_ at 14 DPV and 3.02 log_10_ at 28 DPV) in all goats (Figures 2, 3; Table 1). Starting from 56 DPV, the percentage of VNT-positivity slightly decreased; i.e. 96, 74 and 86% at 56, 150 and 180 DPV, respectively. However, median VNT titres remained good at those timepoints; 2.85 log_10_ at 56 DPV, 2.18 log_10_ at 150 DPV and 2.37 log_10_ at 180 DPV (Figures 4–6, Table 1). All sera collected from double-vaccinated goats showed the ability to neutralise O JORD at 56 DPV with a median of 3.42 log_10_ and 93 and 96% of double-vaccinated goats were still VNT-positive at 150 and 180 DPV, respectively, with a median of 2.53 log_10_ and 2.67 log_10_ (Figures 4–6; Table 1).

#### 3.1.3 Comparison of immunisation against the two strains serotype O

To determine whether the observed variation in median VNT titres between O BFS and O JORD strains was due to random variation, we compared data from goat sera collected at 28 DPV (the standard timepoint for evaluating vaccine-induced immune responses). Statistical analysis (Welch t-test) showed no significant difference between the two strains (t = 0.057, df = 110, *p*-value = 0.9557), confirming that the median titres were statistically comparable.

### 3.2 Sheep

#### 3.2.1 Ability to neutralise the strain “O BFS”

A single vaccine administration resulted in seroconversion of 77% (median VNT titre 2.05 log_10_) and 81% (median titre 2.47 log_10_) of sheep at 14 DPV and 28 DPV, respectively. The level of neutralising antibody in singly vaccinated animals showed a slight decrease starting at 56 DPV, with 79, 56 and 60% of sheep VNT positive at 56, 150 and 180 DPV, respectively, and with median VNT titres of 2.26 log_10_ at 56 DPV, 1.71 log_10_ at 150 and 1.81 log_10_ at 180 DPV (Figures 2–6; Table 1). The 22 sheep that received a booster dose were all VNT positive at 56 and 150 DPV, while only 77% were VNT positive at 180 DPV (Figures 5, 6; Table 1). Medians VNT titres were 3 log_10_ at 56 DPV, 2.34 log_10_ at 150 DPV and 2.11 log_10_ at 180 DPV (Figures 4–6; Table 1).

#### 3.2.2 Ability to neutralise the strain “O JORD”

For the O JORD strain, 76% of sheep were VNT positive at 14 DPV with median VNT titres of 2.16 log_10_ (Figures 2, 3; Table 1). At later timepoints, 73% of sheep were seropositive with a median VNT titre of 2.02 log_10_ at 28 DPV, 66% with a median VNT titre of 2.11 log_10_ at 56 DPV, 53% with a median VNT titre of 1.52 log_10_ at 150 DPV and 53% with a median titre of 1.66 log_10_ at 180 DPV (Figures 2–6; Table 1). All sheep receiving two doses of vaccine were VNT positive until the endpoint of the study (i.e., 180 DPV) with median VNT titres of 3.01 log_10_ at 56 DPV, 2.41 log_10_ at 150 DPV and 2.43 log_10_ at 180 DPV (Figures 4–6; Table 1).

#### 3.2.3 Comparison of immunisation against the two strains serotype O

A statistical comparison of the VNT responses at 28 DPV showed no significant difference between the response of sheep to O JORD and O BFS (*t* = −0.0032, df = 102, *p*-value = 0.9974).

## 4 Discussion

The objective of the present study was to evaluate if the FMD vaccine, chosen for the campaign carried out in Jordan during 2022 and 2023, was effective in inducing an immune response in small ruminants. A post-vaccination monitoring (PVM) study was undertaken to achieve this aim, involving two independent groups of small ruminants, one group of goats and one group of sheep, receiving a commercially available trivalent vaccine, including two FMDV serotypes. This study was conducted to investigate vaccine-induced humoral responses in sheep and goats against a homologous strain and a heterologous strain of FMD serotype O virus to generate data that can be used to corroborate, *in vivo*, vaccine matching results. Similar investigations were previously performed using various vaccines against FMD in other countries (18, 35, 36). After vaccination, the induced immune response was evaluated using virus neutralisation testing (VNT) on sera collected from vaccinated animals at specific timepoints, in order to measure (i) the early immune response, (ii) the response at the presumed peak of immunization and (iii) the persistence, in the vaccinated animals, of neutralising antibodies.

The two populations of small ruminants involved in the present PVM study received the same treatment, in terms of dose of administered vaccine, but they showed differences in the immunological reaction to the vaccination. Goats responded very well against O BFS after the first vaccination; as 98% of the population was already VNT positive after 14 days and at 28 DPV humoral immunity reached the peak level, with all the animals VNT-positive. Neutralising antibodies persisted until 180 DPV, with the percentage of positive animals exceeding the 70% threshold considered necessary for effective vaccination coverage (18, 37). At these timepoints, also the intensity of immune response was good and persistent. The booster vaccination improved both the percentage of positivity (close to 100% from 56 to 180 DPV) and VNT titres (2.63 log_10_–3.4 log_10_). The immune response against O BFS observed in sheep was similar to that described for goats, but with a lower percentage of positivity and lower VNT titres. The highest positivity rate of 81% was observed at 28 DPV, with 2.47 log_10_ as median of VNT titres. Given the requirement for 70% of the population to be immunised for vaccination to be effective, single dose vaccination of sheep could only be considered effective until 56 DPV as only 56 and 62% of sheep were VNT positive at 150 and 180 DPV, respectively. Again, the second administration of vaccine induced a strong booster response in sheep with higher positivity rates (i.e., 100% at 56 DPV and 150 DPV; 77% at 180 DPV) and stronger median VNT titres.

A notable observation was also made, related to magnitude and duration of the serological response to vaccination in sheep and in goats. In fact, the percentage of double-vaccinated sheep that were VNT positive (77%) and the mean VNT titre of those sheep (2.11 log_10_) at 180 DPV, was lower than that of singly-vaccinated goats (86% and 2.34 log_10_ respectively).

The discrepancy observed between goats and sheep during this study was not supported by statistical analysis, but we can speculate that it could be related to different factors, including intrinsic differences between the immune system of the two species or difficulties with vaccine administration under field conditions, but we cannot rule that the different housing of the two groups affected the immunisation. Previous studies demonstrated that ruminant immune responses can vary by species (38, 39) but can also be influenced by environmental factors, such as diet, temperature/climate, stable crowding and housing-related stress (27, 28). Finally, field veterinarians may face also practical challenges when vaccinating sheep with long wool, which could affect dosing and vaccine uptake. All of these suppositions, which lie outside the scope of this study and have not statistically significance, are reported as observations and will be investigated more specifically in the future.

Although the present study was not designed to investigate specie-specific differences in the immunisation, the observed patterns strongly support the conclusion that booster vaccination with this commercial vaccine is necessary to achieve robust and durable immunity in small ruminant populations, particularly in sheep. In fact, double-vaccinated sheep showed higher positivity rate than single-vaccinated animals (100% at 56 and 150 DPV), and their VNT titres at these timepoints are even higher than those observed in single-vaccinated sheep at 28 DPV, the supposed peak of the immune response post vaccination.

The particular focus of this study was on evaluating the possibility that the antibodies arising after vaccination could actually neutralise also a circulating strain of FMD serotype O virus that is still threatening the country, O JORD. This is particularly interesting because the vaccine strain and the Jordan field strain are not phylogenetically close. Previous data about vaccine matching between O/EURO-SA/O_1_/Campos and O/ME-SA/PanAsia-2/ANT-10 demonstrated an overall good antigenic correlation. The strain included in the vaccine formula showed good r_1_ values, namely 0.59 and 0.48, relatable to a good antigenic correlation and to an assumed ability of the vaccine strain to induce cross-protection against that specific field strain (16, 40). Performing VNT with O JORD, a strain of FMD serotype O virus still circulating in the Middle-East area, yielded valuable data.

The study showed that, in both species, immunisation against O BFS, homologous to the vaccine strain, and the heterologous O JORD were very similar. Single vaccinated goats showed good and persistent VNT titres against O JORD after a single vaccination with positivity percentage always higher than 70%, and double vaccinated animals sera showed an improvement in VNT titres and positivity rate, while sheep sera had lower neutralisation antibodies against O JORD, in comparison with goats, in terms of positivity rates and VNT titres. As previously detected against O BFS, immunisation in sheep decreased, falling below the thresholds of VNT positivity rate for effective vaccination (70%) in the latest phases of the study (i.e., 56–180 DPV) but the second administration of vaccine in sheep improved the efficacy of the treatment, increasing the VNT positivity rate and titres.

Overall, the vaccination conducted on animals in the field induced a very similar (almost identical) immunisation against the two FMDV serotype O strains. In fact, considering data from sera collected at the peaks of immunisation (28 DPV), no statistical differences could be detected in sera ability to neutralise O BFS or O JORD, neither in goats nor in sheep.

Although no statistically significant variations were detected between immunisation against the two FMDV strains, it is noteworthy that sera from singly vaccinated animals neutralised the field strain O JORD less effectively than O BFS, especially with respect to duration. This pattern was observed in goats, where the proportion of positive animals at later time points hovered was very close to the 70% threshold, and in sheep, which showed a decrease in positivity below that threshold. These findings underscore again the importance of adhering to the manufacturer’s vaccination schedule, including booster doses, to achieve robust immunisation and improve the likelihood of adequate coverage. These data are worthy of note because they confirmed in the field and *in vivo* the conclusions of vaccine matching conducted *in vitro*, corroborating the preliminary hypothesis that vaccines with high concentration of O/EURO-SA/O_1_/Campos antigen might provide cross-protection from new incursions of O/ME-SA/PanAsia-2/ANT-10 in the region.

Concluding, this study found that a commercial vaccine used for an FMD vaccination campaign in Jordan induced a strong neutralising antibody response in small ruminants – with a better response observed in goats than in sheep. Booster vaccination, as recommended by the vaccine manufacturer, is mandatory to provide and maintain higher and longer-lasting antibody levels. This field trial strongly corroborates the results of vaccine matching, confirming that the vaccine in question is a good candidate for the control of FMDV serotype O in the Middle East.

## Data Availability

The raw data supporting the conclusions of this article will be made available by the authors, without undue reservation.

## Footnotes

1 https://www.foot-and-mouth.org, April 2026

2 https://www.gf-tads.org/fmd/progress-on-fmd-control-strategy/en/, April 2026
